# Predictors of Immunological Failure of Antiretroviral Therapy among HIV Infected Patients in Ethiopia: A Matched Case-Control Study

**DOI:** 10.1371/journal.pone.0115125

**Published:** 2014-12-23

**Authors:** Wondu Teshome, Anteneh Assefa

**Affiliations:** School of Public and Environmental Health, College of Medicine and Health Sciences, Hawassa University, Hawassa, Ethiopia; University of Texas Health Science Center San Antonio Texas, United States of America

## Abstract

**Background:**

In resource constrained settings, immunological assessment through CD4 count is used to assess response to first line Highly Active Antiretroviral Therapy (HAART). In this study, we aim to investigate factors associated with immunological treatment failure.

**Methods:**

A matched case-control study design was used. Cases were subjects who already experienced immunological treatment failure and controls were those without immunological failure after an exactly or approximately equivalent duration of first line treatment with cases. Data were analyzed using SPSS v16.0. Conditional logistic regression was carried out.

**Results:**

A total of 134 cases and 134 controls were included in the study. At baseline, the mean age ±1 SD of cases was 37.5±9.7 years whereas it was 36.9±9.2 years among controls. The median baseline CD4 counts of cases and controls were 121.0 cells/µl (IQR: 47–183 cells/µl) and 122.0 cells/µl (IQR: 80.0–189.8 cells/µl), respectively. The median rate of CD4 cells increase was comparable for the two groups in the first six months of commencing HAART (P = 0.442). However, the median rate of CD4 increase was significantly different for the two groups in the next 6 months period (M_6_ to M_12_). The rate of increment was 8.8 (IQR: 0.5, 14.6) and 1.8 (IQR: 8.8, 11.3) cells/µl/month for controls and cases, respectively (Mann-Whitney U test, P = 0.003). In conditional logistic regressions grouped baseline CD4 count (P = 0.028), old age group and higher educational status (P<0.001) were significant predictors of immunological treatment failure.

**Conclusion:**

Subjects with immunological treatment failure have an optimal rate of immunological recovery in the first 6 months of treatment with first line HAART, but relative to the non-failing group the rate declines at a later period, notably between 6 and 12 months. Low baseline CD4 count, old age and higher educational status were associated with immunological treatment failure.

## Introduction

Treatment with Highly Active Antiretroviral Therapy (HAART) has significantly improved the quality of life and life expectancy of People Living with HIV and AIDS. In 2011, more than 8 million people living with HIV and AIDS in low and middle income countries were receiving HAART [1].

The primary goals of initiating HAART among HIV patients are to suppress HIV viral replication and to restore immune function. The clinical decision to check whether such goals have been achieved is made through periodic viral load testing and/or CD4 cell counting [2]. However, virological monitoring is not done routinely because of its high cost, advanced technological infrastructure requirement and limited access in most resource limited settings [3], [4].

CD4 cell count is a crucial element in monitoring treatment response and practically it remains the best predictor for immunological failure. However, different studies have consistently reported low sensitivity of CD4 counts in detecting virological failure among African adults [3], [5], [6]. This implies that some patients will keep on taking a failed regimen which in turn may result in higher mortality rates as evidenced by a study conducted in Haiti [7].

Even though the definition of immunological failure of therapy is not straight forward, the WHO defined criteria have been widely used. Using these criteria, patients who have experienced immunological failure were found to have an increased risk of clinical progression to AIDS and mortality when compared with patients with complete immunological response [8]–[12]. Treatment switching to second line regimen in Ethiopia is guided by using the WHO's criteria. For instance, according to the 2010 national update on HIV, out of 156, 083 patients who were initiated on first line HAART, around 0.6% (n = 865) of them had their treatment switched to second line therapy because of experiencing treatment failure [13].

Understanding of factors that are associated with immunological treatment failure could be a key step in offering stringent care for those at risk of treatment failure. Several clinical and other factors that contribute to poor immunological response to HAART treatment have been documented. However, most of such factors were from routine clinical documents and lack other socio-demographic variables as potential predictors of immunological failure. Therefore, in this study we aim to investigate clinical, and other socio-demographic variables associated with immunological failure in resource limited setting.

## Methods and Materials

### Study design and setting

A matched case-control study was conducted in two major hospitals which started delivering HIV clinical care services in 2004/5 in Ethiopia; namely Adama Hospital in Oromia Region and Yirgalem Hospital in Southern Nations, Nationalities and Peoples' Region (SNNPR). According to the 2010 report of Ethiopian Federal HIV/AIDS Prevention and Control Office, a total of 10,881 patients have ever started ART and 7,171 patients were actively on treatment in the two hospitals in 2010 [13].

Eligibility criteria for initiating HAART during the study period of interest was: stage I and II when CD4 count <200, stage 3 when CD4 count <350 cells/µl and stage 4 at any CD4 count [2]. Immunological treatment failure is defined as CD4 cells decline by 50% from its peak value, or persistently <100, or a fall of CD4 counts below the baseline count [14]. In this context viral load is not used to monitor response to treatment.

### Data collection procedure and data collectors

Both case and control participants were initially identified from routine ART registers. This register contains basic clinical and a few demographic variables of all subjects who are starting treatment. Normally, the patient's information on this register is updated every time the client comes to the HIV care clinic. In addition to the baseline information, this register also contains the CD4 count, weight and functional status of the clients at the 6^th^ month of initiating treatment and then at every 12 month interval.

Data were collected from follow up forms, ART registers and through direct interview with patients using structured and pretested questionnaire. Information retrieved from follow up forms and ART registers include number of visits paid to the hospitals, date of treatment initiation and date of experiencing immunological treatment failure, weight, height and CD4 counts. Other variables like residential area, marital status, and educational status were obtained through direct interview with the study subjects.

HIV care clinic health workers in both hospitals conducted the data collection with periodic supervision from the investigators. Ethics Committee of Hawassa University approved the study and written consent was received from the study participants.

The primary end-point was immunological treatment failure defined using the WHO criteria. Quantitative rate of increment for CD4 counts was also investigated. Possible determinants for immunological treatment failure were age, place of residence, marital status, baseline CD4 count, regimen type, baseline WHO clinical stage, and average health facility visiting time in months, baseline body mass index (BMI), educational status, and sex.

### Procedure to identify case and control participants

In this study, cases were subjects who experienced immunological treatment failure and controls were those who didn't experience immunological treatment failure after taking first line treatment for an exactly or approximately equivalent duration with cases. Once cases were identified, they were matched with controls who started treatment in the same month and year with the cases. This was achieved because the ART register was filled in such a way that all subjects who started treatment with in same month and year were entered on the same page and they were not mixed up with patients starting treatment in another month of the same year. In case of multiple possible controls, one control was selected using lottery method.

Initially, the ART registers in both hospitals were carefully examined to pick up the required number of cases and controls. On the ART register, each row corresponds to a patient and the second page of the register contains the updated information at different visiting times; namely, at treatment initiation, after 6 month of starting treatment and then every year. Initially, we screened each patient's row to verify whether they had already experienced immunological failure or not. We reviewed the ART registers of patients who started treatment from November, 2004 to June, 2012. After checking a total of 4,000 patient rows, we had identified 170 subjects who experienced immunological treatment failure. However, 36 subjects weren't actively on treatment (were either transferred out or lost from care) and thus 134 subjects who experienced immunological treatment failure were selected as cases (n = 134) and each were paired with single controls, resulting in a total of 268 study subjects. The whole data collection took place from February 2013 to August 2013.

### Data analysis

Data were entered, cleaned and analyzed using SPSS V 16.0. Normality tests were performed before running any statistical computations. CD4 cell count response was calculated using median rate of increase from baseline to 6 months, from 6 months to 12 months and from 12 months to 24 months. Nonparametric test (Mann-Whitney U test) was used to compare CD4 counts in the two groups.

Univariate conditional logistic regression was first used to detect possible predictors of immunological treatment failure. Multivariate analysis was performed by fitting a stratified Cox model on matched pairs to adjust for potential confounders. Variables showing significant effect during univariate analysis and other possible determinant variables from literature reviews were included in the stratified Cox model. Results were reported as the multivariate adjusted odds ratios (AORs) with 95% confidence intervals (CIs) for the association between predictor variables and the outcome (immunological treatment failure). For all statistical tests, significance level was set at alpha (α) of 0.05.

## Results

### Baseline characteristics of cases and controls

At baseline, the mean (±1SD) age of cases was 37.5±9.7 years, whereas it was 36.9±9.2 years among controls. The median baseline CD4 count was 121.0 cells/µl (IQR: 47–183 cells/µl) for cases and 122.0 cells/µl (IQR: 80.0, 189.8 cells/µl) for controls. The median baseline CD4 count wasn't significantly different in the two groups (Mann-Whitney U-test, P = 0.227). At baseline 45.2% (n = 28), 51% (n = 78) of controls and 54.8% (n = 34), 48.7% (n = 74) of cases were in WHO clinical stage IV and III respectively. All study participants (both cases and controls) were started on first-line regimen composed of two nucleoside analogue-based drugs (zidovudine (AZT) or stavudine (d4T) or tenofovir (TDF) plus lamivudine (3TC)) plus one non-nucleoside analogue drug (either nevirapine (NVP) or efavirenz (EFV)). D4T/3TC/NVP was the commonly initiated regimen among both cases and controls. Other baseline characteristics of study participants are shown in table 1.

**Table 1:** Distribution of different variables for cases and controls participants.

*Variable*		*Immunologic failure*	
		*Yes (%)*	*No (%)*
***Family size, Mean persons ± SD***		4.2±2.5	3.9±2.4
***Age, Mean years ± SD***		36.5±9.7	39.0±10.3
***Place of residence***	Urban	103 (76.9)	97 (72.4)
	Rural	31(23.1)	37 (27.6)
***Educational status***	Illiterate	14 (10.5)	27 (20.1)
	Able to read and write only	69 (51.5)	73 (54.5)
	Grade 1–12	27 (20.1)	15 (11.2)
	Tertiary level education	24 (17.9)	19 (14.2)
***Sex***	Male	61 (45.5)	65 (48.5)
	Female	73 (54.5)	69 (51.5)
***Marital status***	Currently not married	68 (50.7)	63 (47.0)
	Married	66 (49.3)	71 (53.0)
***Baseline CD4 count, cells/micro litre***	0–49	32 (25.4)	16 (12.5)
	50–99	22 (17.5)	28 (21.9)
	100–149	20 (15.9)	35 (27.3)
	150–249	38 (30.1)	30 (23.4)
	> = 250	14 (11.1)	19 (14.8)
***Median (IQR)***		***121(47, 183)***	***122 (80.0, 189.9)***
***WHO clinical stage***	Stage 1 or 2	26 (19.4)	28 (20.9)
	Stage 3	74 (55.2)	78 (58.2)
	Stage 4	34 (25.4)	28 (20.9)
***BMI, baseline***	<18.5	55 (41.0)	49 (36.6)
	> = 18.5	79 (59.0)	85 (63.4)
***Original HAART regimen***	D4T based	73 (54.5)	70 (52.2)
	AZT based	41 (30.6)	46 (34.3)
	TDF based	20 (14.9)	18 (13.4)
***First regimen (NNRTIs)***	NVP based	96 (71.6)	100 (74.6)
	EFV based	38 (28.4)	34 (25.4)
***Average health facility visiting time (months)***	0.8–2.0	36 (27.0)	29 (21.6)
	2.01–3.0	49 (36.5)	63 (47.0)
	>3	49 (36.5)	42 (31.4)
***Total (similar for all sub groups)***		***134 (50.0)***	***134(50.0)***

### Follow-up status

Median follow up duration was 57.5 months (IQR: 35.5–75.2 months) for controls. Similarly, cases had median follow up duration of 59 months (IQR: 38.5–76.0 months). This comparable follow up duration (Mann-Whitney u-test, p-value  = 0.681) was achieved by selecting controls who were initiated on HAART in similar month and year with cases.

### Switching time and rate of CD4 increase

The median switching time from first line to second line HAART was 25.0 months (IQR: 14.0–44.0 months). The common second line regimes were Abacavir/Didanosine/Kaletra (ABC/DDI/Kaletra) and TDF/3TC/Kaletra. CD4 counts were consistently recorded in the first 2 years. The third, fourth and fifth year CD4 counts were highly affected by missing values and were excluded from rate calculations.

CD4 cell counts poorly fit normality assumptions for both cases and controls (Kolmogorov-Smirnov test, P<0.001). Moreover, different transformations tried couldn't fulfill normality assumptions.

**Figure 1:**
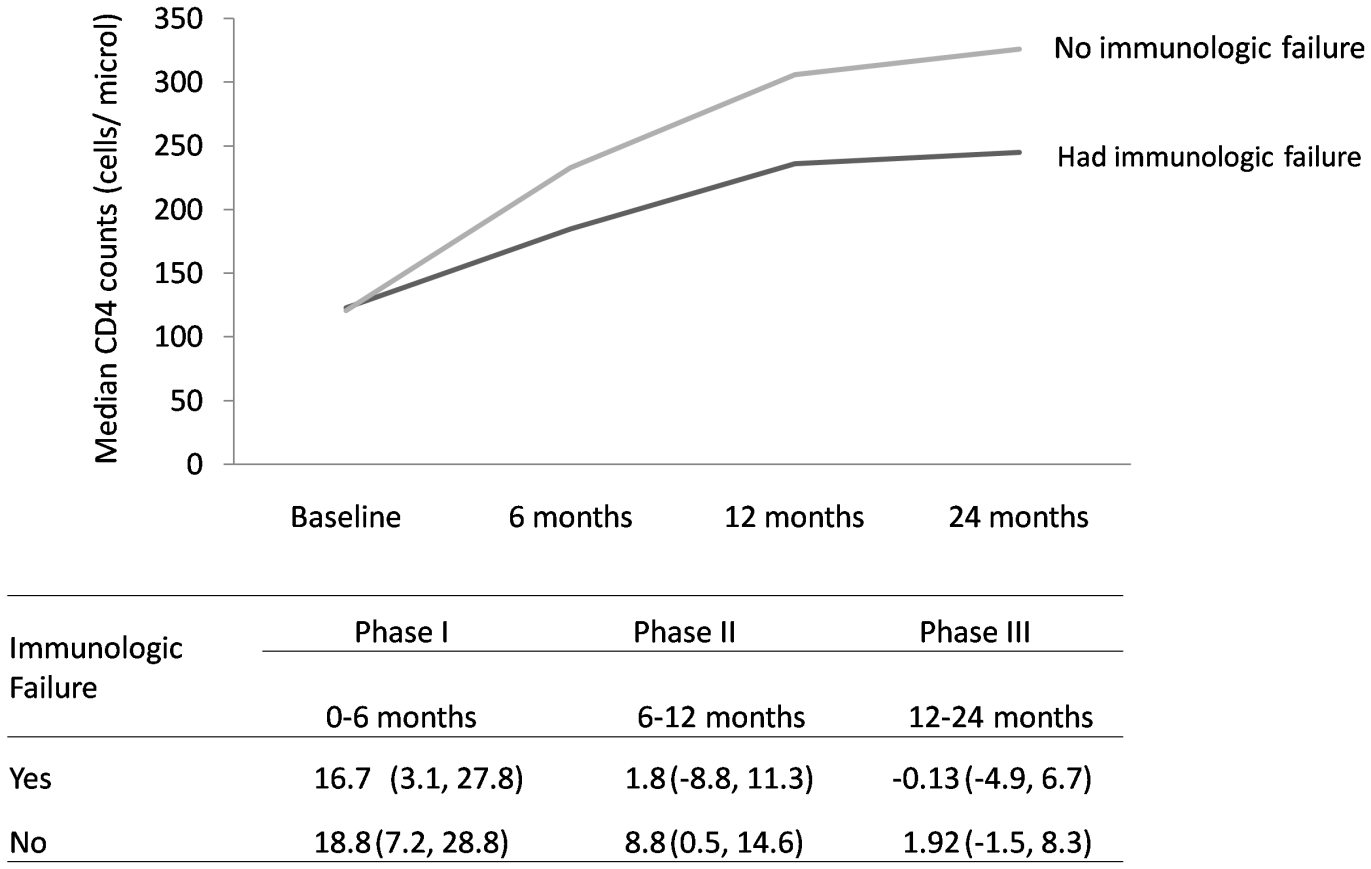
Graph showing the median CD4 count at baseline and at different periods of treatment, stratified by immunologic failure status. The table below the graph indicates the median rate of CD4 increase (cells/µl/month) between the different periods and its IQR.

The median rate of increase was calculated for subjects who had CD4 counts between the two given points namely month0 to month6, month6 to month12 and month12 to month 24. Accordingly, the number of participants who had documented CD4 counts between month0 to month6, month6 to month12 and month12 to month24 were 94, 77 and 87 for the immunologically non-failed group and they were 92, 82 and 84 among the immunologically failed group respectively. The median rate of CD4 cells increase was almost similar for the two groups in the first six months of commencing HAART (18.8 and16.7 cells/µl/month for controls and cases respectively, Mann-Whitney U test, P = 0.442). The median rate of CD4 increase was significantly different for the two groups for the next 6 months period. The rate of increment was 8.8 (IQR: 0.5, 14.6) and 1.8 (IQR: −8.8, 11.3) cells/µl/month for controls and cases, respectively (Mann-Whitney U test, P = 0.003). In the second year (12 month to 24 months) the rate of increase slowed down remarkably for the two groups (1.92 and −0.13 cells/µl/month for controls and cases) and wasn't significantly different among the two groups (Mann-Whitney U test, P = 0.116) (Fig. 1).

The prevalence of baseline CD4 count of <250 cells/µl among control subjects was 85.0%, and 89.0% among case subjects; this prevalence has declined to 53.0% for controls and 65.0% for cases after 6 months of treatment. The prevalence of CD4 counts of <50, 50–99, 100–149, 150–249 and ≥250 after 6 months of treatment was 8.2%, 6.1%, 24.5%, 26.5% and 34.5% among cases and was 2.0%, 5.1%, 10.2%, 35.7%, and 46.9% among controls respectively.

### Clinical factors affecting immunological treatment failure

Both conditional univariate and multivariate analysis were carried out to assess potential predictors of immunological treatment failure. In the conditional univariate analysis, baseline CD4 count significantly predicts immunological treatment failure. The odds ratio of immunological failure was 0.27 (95%CI 0.09, 0.81) times less likely among the > = 250 cells/µl CD4 cell count when compared with subjects with baseline CD4 count of <50 cells/µl. The adjusted analysis also remained in the same direction. The average health facility contact time (in months) was also assessed as potential predictor and was found that clients who had infrequent visits to visit health facilities were found to have lesser odds of experiencing treatment failure but this was not statistically significant (Table 2).

**Table 2:** Univariate and multivariate analysis of clinical predictors of immunologic treatment failure.

*Variables*		*Unadj OR (95% CI)*	*Adj OR (95% CI)*
***Baseline CD4 count, cells/µl***	0–49	Ref.	Ref.
	50–99	0.36 (0.15, 0.83)*	0.31 (0.12, 0.78)*
	100–149	0.29 (0.13, 0.68)*	0.23 (0.09, 0.58)*
	150–249	0.55 (0.23, 1.34)	0.44 (0.17, 1.17)
	> = 250	0.30 (0.11, 0.82)*	0.27 (0.09, 0.81)*
***BMI, Kg/m2***	<18.5	Ref.	
	> = 18.5	0.84 (0.51, 1.41)	0.95 (0.53, 1.72)
***WHO stage***	1 or 2	Ref.	Ref.
	3	1.04 (0.54, 2.00)	1.19 (0.54, 2.62)
	4	1.34 (0.64, 2.92)	0.77 (0.30, 1.97)
***Average health facility visiting time (months)***	0.8–2.0	Ref.	Ref.
	2.01–3.0	0.58 (0.27, 1.26)	0.48 (0.18, 1.26)
	>3	0.85 (0.36, 2.01)	0.67 (0.25, 1.80)
***First line regimen (NRTIs)***	D4T based	Ref.	Ref.
	AZT based	0.88 (0.49, 1.59)	0.60 (0.29, 1.22)
	TDF based	1.11 (0.45, 2.76)	1.02 (0.35, 3.01)
***First regimen (NNRTIs)***	NVP	Ref.	Ref.
	EFV	1.14 (0.69, 1.90)	1.21 (0.65, 2.25)

* statistically significant, OR =  Odds Ratio, Unadj =  Unadjusted, Adj =  Adjusted, Ref. =  Reference category.

### Sociodemographic factors affecting immunological treatment failure

In the conditional univariate analysis, baseline age was found to be a significant predictor of immunological treatment failure. The odds ratio of immunological failure was 1.78 (95%CI 1.03, 3.50) times more likely among the older age group (≥40 years) than younger ones (<40 years). The adjusted analysis also remained in the same direction. Moreover, subjects with higher educational status were found to have higher odds of experiencing immunological treatment failure. For instance, patients who have attained tertiary level education were 3.51 times more likely to experience immunological treatment failure when compared with those who have never attended formal education (Table 3).

**Table 3:** Univariate and multivariate analysis of socio-demographic predictors of immunologic treatment failure.

*Variables*		*Unadj OR (95% CI)*	*Adj OR (95% CI)*
***Age, years***	<40	Ref.	Ref.
	> = 40	1.78 (1.03, 2.83)*	1.93 (1.07, 3.50)*
			
***Sex***	Male	Ref.	Ref.
	Female	1.05 (0.67, 1.65)	0.81 (0.47, 1.42)
***Educational status***	No formal education	Ref.	Ref.
	Able to read and write only	1.48 (0.67, 3.24)	1.44 (0.64, 3.25)
	Attended formal education	5.74 (1.72, 19.14)*	6.18 (1.78, 21.42)*
	Tertiary	3.51 (1.07, 11.50)*	3.69 (1.07, 12.68)*
***Place of residence***	Urban	Ref.	Ref.
	Rural	0.65 (0.32, 1.31)	0.93 (0.43, 2.00)
***Marital status***	Currently not married	Ref.	Ref.
	Married	0.87 (0.52, 1.46)	0.79 (0.45, 1.39)

* statistically significant, OR =  Odds Ratio, Unadj =  Unadjusted, Adj =  Adjusted, Ref. =  Reference category.

## Discussion

Retaining patients on first line ART treatment for as long as possible is one strategy to maximize the benefits gained from first line regimens which are cheap and easy to take for patients compared with second line regimen [2].

The median switching time observed in this study was 25.0 months (IQR: 14.0–44.0 months) and this is consistent with findings elsewhere [6], [15], [16]. It was observed that the immunologically failing and non-failing groups had relatively higher median rate of CD4 cells increase in the first six months of treatment. A slightly higher rate of increase was observed for control group than for cases, however, the difference failed to attain level of statistical significance. This phenomenon of first phase acceleration in the rate of CD4 increment was observed in other studies [15], [17].

In the next phase, month 6 to month 12, the CD4 median rate of increase has shown remarkable difference in the two groups. The rate of increment was 4.9 times higher among controls when compared with cases. The seed of treatment failure might have already been sown at this stage even though the median switching time was 25 months (1^st^ quartile1 = 14 months), long after warning sign. This finding may suggest rate of CD4 increment in this period might be used to predict future immunological failure. Among patients with CD4 count of <50 cells/µl, 66.7% of them experienced immunological treatment failure at later time. Timing of immunological treatment failure also depended on the baseline CD4 count. For instance, out of 36 cases of treatment failure in the first 14 weeks on treatment initiation, 77.8% (n = 28) had CD4 counts of <200 cells/µl. The effect of baseline CD4 count was also significant on conditional multiple logistic regression after controlling for possible measured confounders. The probability of developing new opportunistic infections like tuberculosis is high if the baseline CD4 count is low [17], [18] which in turn might contribute to the poor rate of CD4 cells recovery. This effect of baseline CD4 counts on immunological recovery and hence treatment switching has been consistently reported from several studies [18]–[22].

Another major predictor of immunological failure which would have been considered was adherence to treatment as such observations were made in other studies [6], [23]. Adherence is well known to affect not only immunological response to treatment but also morbidity and probability of survival. To achieve a good clinical response and to delay switching of treatment, 95% level of adherence to HAART is required [24], [25]. However, as we had retrospectively followed patients for long period of time, we found it very challenging to measure individual patient's adherence level. As a proxy we calculated the average time at which patients visited the hospitals either for picking drugs up or for other purposes. It was found that patients who had a frequent visit had an increased odds ratio of experiencing immunological failure though it wasn't statistically significant. This might have happened because patients with poor treatment response are more likely to have frequent visits than stable patients.

Age was shown to have a significant influence on probability of immunological treatment failure in the univariate analysis and multivariate analysis. In fact, most literatures support that older age groups are more likely to have failure of therapy and switch to second line regimen than younger group [6], [11], [22], [26], [27]. This effect of age on immune recovery and thus switching is due to the associated factor of a decrease in thymic function and other regenerative mechanisms that could impair immune recovery [22]. Viard and colleagues have found the same evidence on a large study conducted to assess the influence of age on immune recovery [28]. The effect of BMI on immunological treatment failure wasn't validated in this study though the point estimate gave a slight decrease among patients with higher BMI. This was inconsistent with findings by Koethe et al [29].

The other significant observation found in this study was the negative association between educational status and immunological recovery. One logical step to explain this will be to go one step behind and look at how educated people do generally in terms of adherence to treatment when compared with less educated people. On the literature we have found no consistent finding addressing this issue. A systematic review conducted by Peltzer et al has found a negative association between educational status and adherence to treatment in 8.2% of the studies [30]. Another paper by Hansana et al has found a negative association between educational status and adherence to treatment [31]. This signals a need for further research in the current context on how educated patients generally do in terms of adherence to treatment.

The limitation of this study could be that the classification of cases ad controls and most of the data were collected from formats and registers which could result in information biases. However, due to the vertical program and rigorous monitoring system of HIV services in both hospitals, such bias might not play an overriding role. In addition, lack of information about those cases who were lost from care may also adversely influence the findings. Using only immunologic criteria may not adequately define treatment failure as immunologic criteria isn't highly sensitive to detect treatment failure. There are documented evidences that there could be virologic response but immunologic response may not be detected.

## Conclusion

In the current study, it was found that patients with low baseline CD4 count, old age group and those with higher educational status are more likely to have immunological treatment failure. It was also noted that patients with immunological treatment failure have an optimal rate of immune recovery in the first 6 months of treatment with first line HAART but relative to the non-failing group the rate significantly decreases at a later period, notably between 6 months and 12 months. Median rate of CD4 cells recovery in the period between 6 and 12 months of first line treatment was significantly different in the two groups. Though its validation might require a large study, it could be a potential area for predicting immunological treatment failure in resource limited setting, where viral load testing is not routinely done.
