# Correction: Predictors of Immunological Failure of Antiretroviral Therapy among HIV Infected Patients in Ethiopia: A Matched Case-Control Study

**DOI:** 10.1371/journal.pone.0128393

**Published:** 2015-05-11

**Authors:** Wondu Teshome, Anteneh Asefa

The second author’s name is spelled incorrectly. The correct name is: Anteneh Asefa. The correct citation is: Teshome W, Asefa A (2014) Predictors of Immunological Failure of Antiretroviral Therapy among HIV Infected Patients in Ethiopia: A Matched Case-Control Study. PLoS ONE 9(12): e115125. doi:10.1371/journal.pone.0115125
